# Cross talk between EBV and telomerase: the role of TERT and NOTCH2 in the switch of latent/lytic cycle of the virus

**DOI:** 10.1038/cddis.2015.145

**Published:** 2015-05-28

**Authors:** S Giunco, A Celeghin, K Gianesin, R Dolcetti, S Indraccolo, A De Rossi

**Affiliations:** 1 Section of Oncology and Immunology, Department of Surgery, Oncology and Gastroenterology, University of Padova, Padova, Italy; 2 Cancer Bio-Immunotherapy Unit, CRO-IRCCS, National Cancer Institute, Aviano, Italy; 3 Immunology and Molecular Oncology Unit, Istituto Oncologico Veneto (IOV)-IRCCS, Padova, Italy

## Abstract

Epstein–Barr virus (EBV)-associated malignancies, as well as lymphoblastoid cell lines (LCLs), obtained *in vitro* by EBV infection of B cells, express latent viral proteins and maintain their ability to grow indefinitely through inappropriate activation of telomere-specific reverse transcriptase (TERT), the catalytic component of telomerase. Our previous studies demonstrated that high levels of TERT expression in LCLs prevent the activation of EBV lytic cycle, which is instead triggered by TERT silencing. As lytic infection promotes the death of EBV-positive tumor cells, understanding the mechanism(s) by which TERT affects the latent/lytic status of EBV may be important for setting new therapeutic strategies. BATF, a transcription factor activated by NOTCH2, the major NOTCH family member in B cells, negatively affects the expression of *BZLF1*, the master regulator of viral lytic cycle. We therefore analyzed the interplay between TERT, NOTCH and BATF in LCLs and found that high levels of endogenous TERT are associated with high NOTCH2 and BATF expression levels. In addition, ectopic expression of TERT in LCLs with low levels of endogenous telomerase was associated with upregulation of NOTCH2 and BATF at both mRNA and protein levels. By contrast, infection of LCLs with retroviral vectors expressing functional NOTCH2 did not alter *TERT* transcript levels. Luciferase reporter assays, demonstrated that TERT significantly activated *NOTCH2* promoter in a dose-dependent manner. We also found that NF-*κ*B pathway is involved in TERT-induced *NOTCH2* activation. Lastly, pharmacologic inhibition of NOTCH signaling triggers the EBV lytic cycle, leading to the death of EBV-infected cells. Overall, these results indicate that TERT contributes to preserve EBV latency in B cells mainly through the NOTCH2/BAFT pathway, and suggest that NOTCH2 inhibition may represent an appealing therapeutic strategy against EBV-associated malignancies.

Epstein–Barr virus (EBV), a human herpesvirus with potent B-cell transforming activity *in vitro*, is linked to a number of B-cell malignancies *in vivo*.^1^ EBV infection transforms human primary resting B lymphocytes into actively proliferating cells that may generate immortalized lymphoblastoid cell lines (LCLs). LCLs are an *in vitro* model of EBV-driven B-cell malignancies, such as post-transplant lymphoproliferative disorders and non-Hodgkin lymphomas. EBV-associated B-cell malignancies and LCLs express latent viral proteins and maintain their ability to grow indefinitely through inappropriate activation of telomerase.^2, 3, 4^

Telomerase is a ribonucleoprotein complex containing an internal RNA template and a catalytic protein with telomere-specific reverse transcriptase activity (TERT) that maintains telomeres at the ends of eukaryotic chromosomes, thus preventing cell senescence and apoptosis.^5, 6^ Recent studies have suggested that, besides maintenance of telomere length, TERT is involved in several other cell functions.^7, 8^ Our previous studies have demonstrated that TERT expression has an important role in preventing the EBV lytic cycle in LCLs, thereby favoring the induction and maintenance of EBV latency in primary B lymphocytes, a prerequisite for EBV-driven transformation. Indeed, high levels of endogenous TERT or ectopic TERT expression in telomerase-negative EBV-infected cells prevent viral lytic cycle induction. By contrast, TERT silencing by specific siRNA or short-hairpin (sh) RNA induces the expression of BZLF1, EBV early antigen diffuse (EA-D) and glycoprotein 350 (gp350) EBV lytic proteins and triggers a complete lytic replication of the virus. This occurs in both EBV-immortalized LCL and fully transformed EBV-positive Burkitt lymphoma (BL) cell lines, thus supporting the concept that TERT is a critical regulator of the balance between EBV latency and lytic replication in B cells.^3, 9, 10^ The fine mechanisms by which TERT level modulates the expression of EBV lytic proteins are still unclear. According to our previous findings, activation of the EBV lytic cycle triggered by TERT inhibition may depend on modulation of BATF, a negative regulator of BZLF1, the main inducer of the viral lytic cycle.^9^ BATF is a transcription factor mainly expressed in hematopoietic tissues and in B cells infected with EBV.^11, 12, 13^ Interestingly, BATF is a target gene of NOTCH signaling in B cells.^13^ The NOTCH gene family encodes transmembrane receptors that modulate differentiation, proliferation and apoptotic programs in response to extracellular stimuli.^14, 15, 16, 17^ NOTCH signaling is activated by the interaction of the extracellular domain of NOTCH with one of its ligands, belonging to the delta-like and jagged families. This interaction induces a conformational change in NOTCH, resulting in two proteolytic cleavages mediated by ADAM protease and gamma-secretase, and cytoplasmic release of the NOTCH intracellular domain (NOTCH-ICD), allowing its translocation to the nucleus, where it participates in transcriptional regulation of target genes.^18^

In particular, NOTCH2 has an important role in the development of marginal zone B cells,^19^ and *NOTCH2* gene mutations or overexpression can be detected in B-cell malignancies.^20, 21, 22, 23, 24, 25, 26, 27, 28, 29, 30^ These observations, together with the demonstration that NOTCH2 can induce the expression of BATF,^13^ prompted us to examine the possible involvement of NOTCH2 in the mechanisms underlying the regulation of EBV latent/lytic status affected by TERT in LCLs. As viral lytic replication is associated with the death of infected cells, discovering the pathways involved in the mechanisms by which TERT regulates the balance between EBV latency and lytic replication may be useful in designing new strategies to treat EBV-driven malignancies.

## Results

### BATF and NOTCH2 are expressed at high levels in TERT-positive LCLs

**Figure 1:**
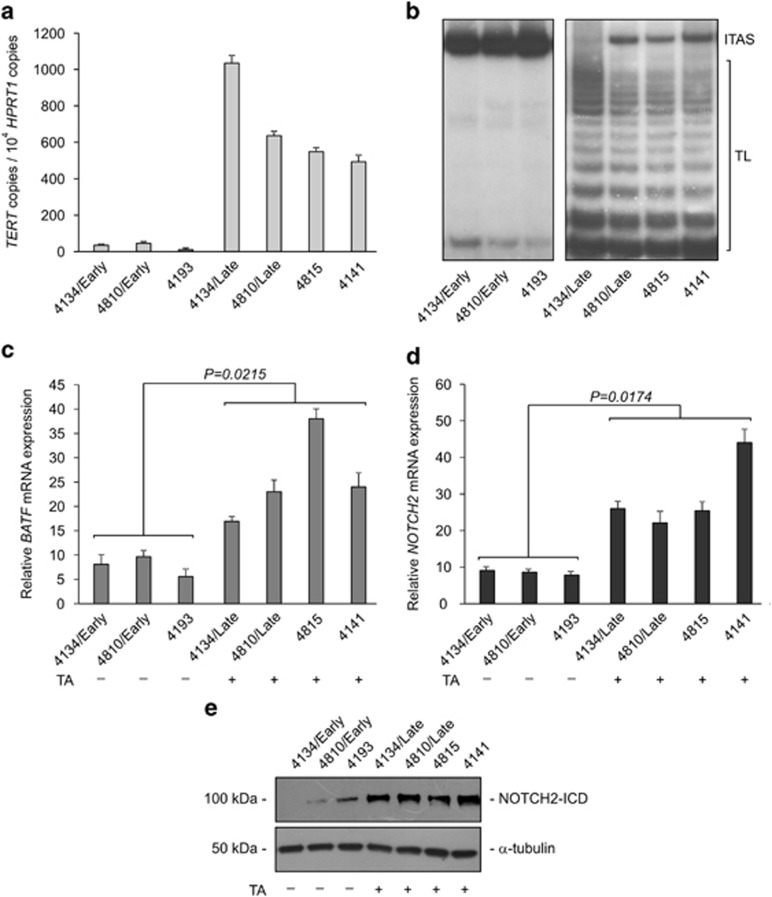
High levels of TERT are associated with high levels of BATF and NOTCH2 expression in LCLs. *TERT* levels, telomerase activity (TA), *BATF* and *NOTCH2* expression in several LCLs. (**a**) *TERT* transcripts were quantified by real-time PCR. Values are means and S.D. (bar) of three replicates. (**b**) TA was analyzed by TRAP assay. TL, telomerase ladder; ITAS, internal telomerase assay standard. Panels are representative of three separate analyses. (**c**) *BATF* and (**d**) *NOTCH2* transcripts were quantified by real-time PCR. Relative units were calculated according to 2^−ΔCt^ formula, with *HPRT1* as housekeeping gene. Values are means and S.D. (bar) of three replicates. (**e**) Expression of NOTCH2 and housekeeping *α*-tubulin proteins was assessed by western blot. Panels are representative of three separate analyses. Presence (+) or absence (−) of TA in cell cultures is reported below

We first examined the expression of *BATF* and *NOTCH2* in LCLs expressing various levels of endogenous TERT. LCLs greatly differed in their timing of TERT expression and telomerase activation; in fact, they exhibit telomerase activity (TA) since their early culture passages after EBV infection or else become telomerase positive during their establishment in culture.^2, 3, 4^ Figures 1a and b show seven representative LCLs with low (4134/Early, 4810/Early and 4193) or high (4134/Late, 4810/Late, 4815 and 4141) levels of endogenous TERT and TA; LCLs with low/undetectable TERT levels and TA had significantly lower *BATF* mRNA levels (*P*=0.0215; Figure 1c). TERT-positive LCLs also had more than threefold higher *NOTCH2* transcript levels than TERT-negative LCLs (*P*=0.0174; Figure 1d). Western blot analysis showed higher levels of NOTCH2 protein in TERT-positive LCLs, thus supporting results of the transcript analysis (Figure 1e). Overall, these findings suggest a possible interplay between TERT, BATF and NOTCH2 levels in LCL cells.

### Ectopic expression of TERT is followed by increased expression of functional NOTCH2 protein

**Figure 2:**
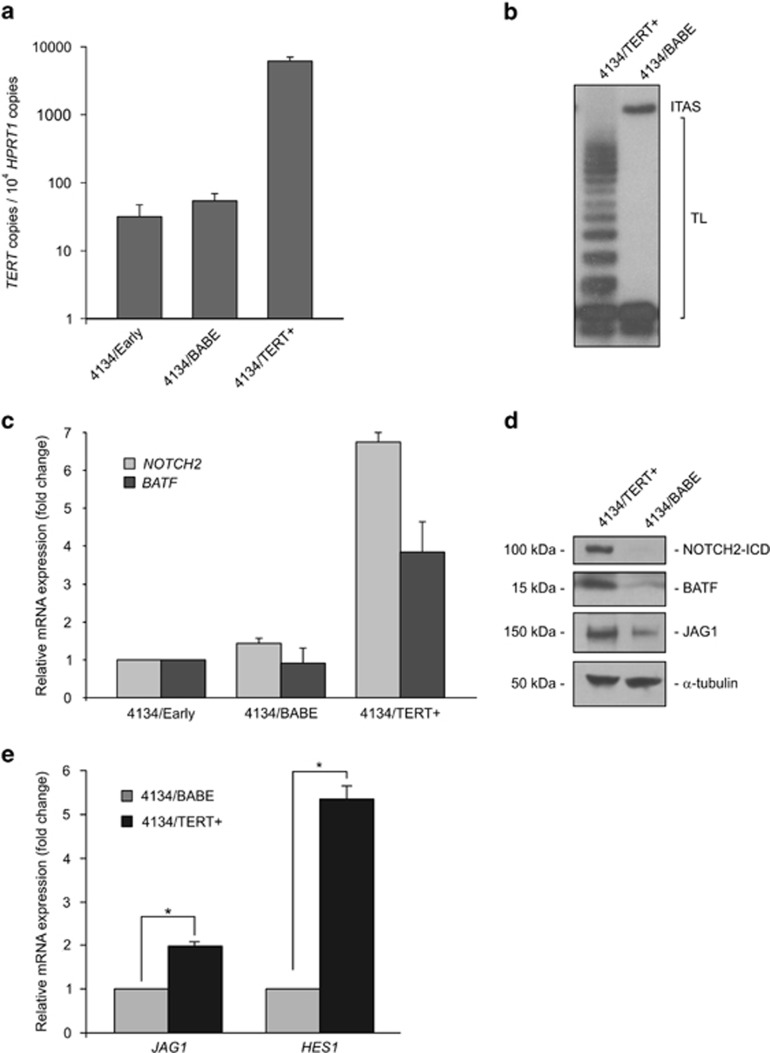
Ectopic expression of TERT induces expression of functional NOTCH2 protein. 4134/Early cells were infected with retroviral vector expressing TERT (4134/TERT+) or with control empty vector (4134/BABE). (**a**) *TERT* transcripts were quantified by real-time PCR. Values are means and S.D. (bar) of three replicates. (**b**) Telomerase activity was tested by TRAP assay. TL, telomerase ladder; ITAS, internal telomerase assay standard. Panel is representative of three separate analyses. (**c**) *NOTCH2* and *BATF* transcripts were quantified by real-time PCR. Values are the means and S.D. (bar) of three replicates. (**d**) Expression of NOTCH2, BATF, JAG1 and housekeeping *α*-tubulin proteins was assessed by western blot. Panels are representative of three separate analyses. (**e**) *JAG1* and *HES1* transcripts were quantified by real-time PCR. Values are means and S.D. (bar) of three replicates. *P<0.05

To investigate whether TERT could modulate NOTCH2 expression, we examined 4134/Early cells transfected with a retroviral vector containing TERT (4134/TERT+) or with the control vector (4134/BABE); 4134/TERT+ cells expressing ectopic TERT showed much higher levels of TERT transcripts than parental or control 4134/BABE cells (Figure 2a). Increased *TERT* mRNA levels were paralleled by a concomitant increase in TA (Figure 2b). Cells expressing ectopic TERT showed increased levels of both NOTCH2 and BATF transcripts and relative proteins (Figures 2c and d). Interestingly, forced TERT expression was accompanied by a significant increase in transcripts and protein expression of jagged 1 (JAG1), one of the NOTCH ligands (Figures 2d and e). The expression of NOTCH2 and JAG1 in 4134/TERT+ cells was also associated with increased NOTCH signaling, as indicated by upregulation of transcripts of hes family bHLH transcription factor 1 (*HES1*), a canonical target gene activated by the NOTCH pathway (Figure 2e). NOTCH signaling in our LCL model is probably activated by interactions between cells expressing both JAG1 and NOTCH2, which may occur in the tight clumps typically formed in B cells after EBV infection.^31, 32^ Upregulation of JAG1 and *HES1* expression was also detected in cells expressing high levels of endogenous *TERT* transcripts (data not shown).

### NOTCH2-ICD does not induce TERT expression

**Figure 3:**
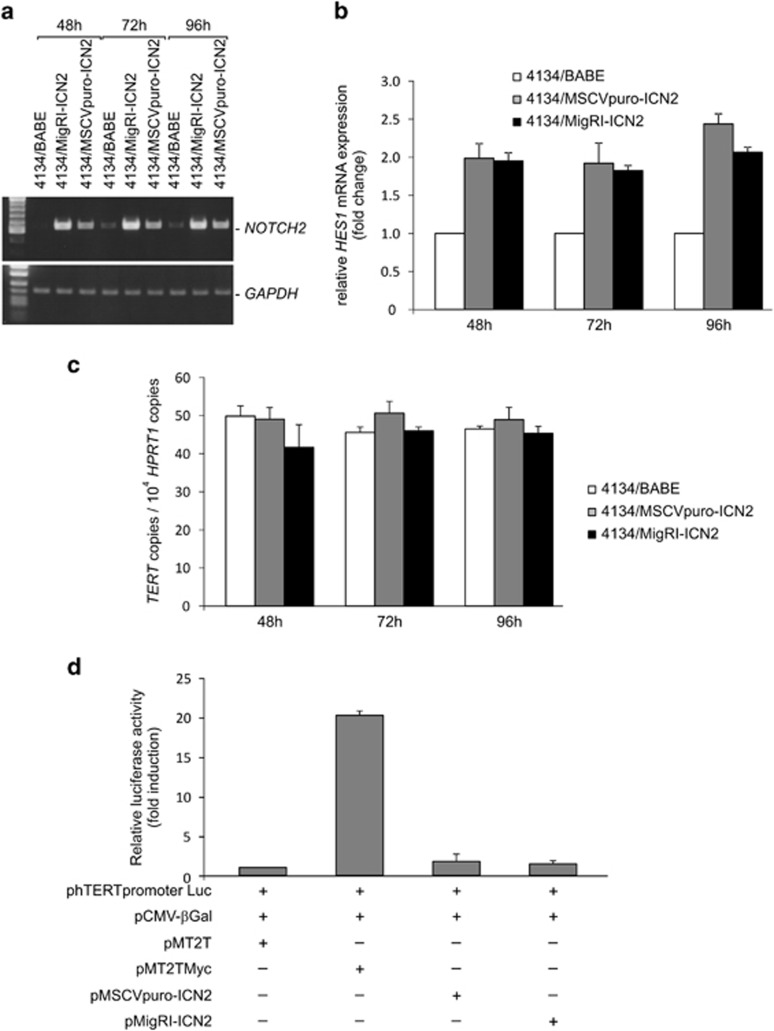
NOTCH2 does not induce TERT expression. 4134/Early cells were infected with retroviral vectors expressing NOTCH2-ICD (4134/MigRI-ICN2 or 4134/MSCVpuro-ICN2) or with control vector (4134/BABE), and analyzed at indicated hours (h) post infection for *NOTCH2, HES1* and *TERT*. (**a**) *NOTCH2* (upper panel) and housekeeping *GAPDH* (lower panel) mRNA were analyzed by RT-PCR. Panels are representative of three separate analyses. (**b**) *HES1* transcripts were quantified by real-time PCR. Values are means and S.D. (bar) of three replicates. (**c**) *TERT* transcripts were analyzed by real-time PCR. Values are means and S.D. (bar) of three replicates. (**d**) U2OS cells were co-transfected with a plasmid expressing luciferase under the control of the *TERT* promoter (phTERTpromoterLuc) and with vectors expressing MYC (pMT2TMyc) or NOTCH2-ICD (pMigRI-ICN2 or pMSCVpuro-ICN2) or with control vector (pMT2T). A plasmid expressing bacterial *β*-Gal gene (pCMV-*β*Gal) was employed as an internal control for transfection efficiency. Luciferase assay was performed 72 h post transfection. Values are means and S.D. (bar) of three separate experiments

To ascertain whether NOTCH2 could also induce TERT expression, 4134/Early cells were infected with a retroviral vector expressing the functional NOTCH2-ICD (4134/MigRI-ICN2 or 4134/MSCVpuro-ICN2) or with a control vector (4134/BABE). *NOTCH2* overexpression in infected cells was confirmed by PCR amplification (Figure 3a) and, as expected, was followed by increased expression of the NOTCH target gene *HES1* (Figure 3b). As shown in Figure 3c, ectopic expression of *NOTCH2-ICD* did not significantly modify the level of *TERT* transcripts. Since NOTCH2 participates directly in transcriptional regulation of nuclear genes, we performed additional experiments to confirm that NOTCH2 did not activate *TERT* expression. TERT-negative U2OS cells were co-transfected with phTERTpromoterLuc, expressing luciferase under the control of the *TERT* promoter, and a plasmid expressing NOTCH2-ICD (pMigRI-ICN2 or pMSCVpuro-ICN2), or a plasmid expressing v-myc avian myelocytomatosis viral oncogene homolog (MYC; pMT2TMyc), a well-known activator of TERT promoter. Luciferase analysis showed that, unlike MYC, NOTCH2-ICD does not activate the *TERT* promoter (Figure 3d).

### TERT transactivates the NOTCH2 promoter via NF-*κ*B signaling

**Figure 4:**
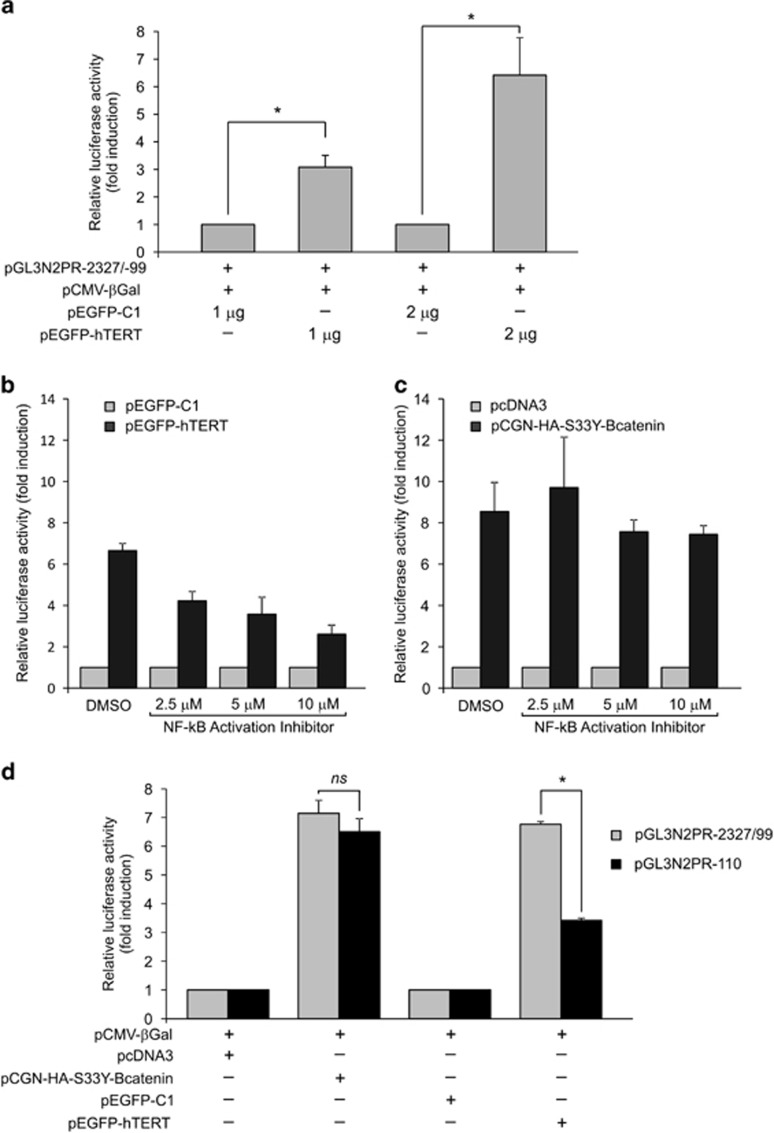
Transcriptional activation of NOTCH2 promoter by TERT. (**a**) HCT116 cells were co-transfected with a plasmid expressing luciferase under control of *NOTCH2* promoter (pGL3N2PR-2327/99) and with 1 or 2 *μ*g of plasmid expressing TERT (pEGFP–hTERT) or control vector (pEGFP-C1). (**b**) HCT116 cells were co-transfected with *NOTCH2* reporter plasmid pGL3N2PR-2327/99 and with pEGFP–hTERT or control pEGFP-C1. Three hours before co-transfection cells were treated with indicated concentrations of N-AI or with DMSO as control. (**c**) HCT116 cells were co-transfected with *NOTCH2* reporter plasmid pGL3N2PR-2327/99 and with a plasmid encoding an S33Y-mutated *β*-catenin protein (pCGN-HA-S33Y-*β*catenin) or a control plasmid (pcDNA3). Three hours before co-transfection cells were treated with indicated concentrations of N-AI or with DMSO as control. (**d**) HCT116 cells were co-transfected with vectors allowing the expression of S33Y-*β*-catenin (pCGN-HA-S33Y-*β*catenin) or TERT (pEGFP–hTERT) or with control vectors (pcDNA3 and pEGFP-C1) and with a plasmid expressing luciferase under control of larger *NOTCH2* promoter (pGL3N2PR-2327/99) or smaller *NOTCH2* promoter (pGL3N2PR-110). A plasmid expressing bacterial *β*-Gal (pCMV-*β*Gal) gene was also co-transfected in each experiment as internal control for transfection efficiency. Luciferase assay was performed 24 h post transfection. Values are means and S.D. (bar) of four separate experiments. NS, not significant; *P<0.05

The finding that TERT overexpression increased *NOTCH2* mRNA levels prompted us to ascertain whether the *NOTCH2* promoter could be activated by TERT. HCT116 cells were co-transfected with the pGL3N2PR-2327/-99 plasmid, carrying the *luciferase* gene under the control of *NOTCH2* promoter^33^ and with pEGFP–hTERT, a plasmid encoding a enhanced green fluorescent protein (EGFP)–TERT fusion protein, or the pEGFP-C1 vector as a control.^34^ Luciferase analysis disclosed that the *NOTCH2* promoter was significantly activated by ectopic TERT expression in a dose-dependent manner (Figure 4a).

Recent studies have indicated a telomere-independent role for TERT as a transcriptional modulator of the Wnt and NF-*κ*B signaling pathways.^35, 36, 37^ Therefore, in order to identify the mechanism exploited by TERT to induce *NOTCH2* expression, we examined the effects of Wnt and NF-*κ*B inhibitors on the regulation of the *NOTCH2* promoter by TERT. HCT116 cells were co-transfected with pGL3N2PR-2327/-99 and pEGFP–hTERT or with pGL3N2PR-2327/-99 and pEGFP-C1 as control, and cultured in serial dilutions of the NF-*κ*B signaling inhibitor NF-*κ*B activation inhibitor (N-AI) or dimethylsulfoxide (DMSO). Luciferase reporter assay performed 24 h after co-transfection showed that N-AI treatment reduced *NOTCH2* promoter activation by TERT in a dose-dependent manner compared with DMSO-treated cells (Figure 4b). To ascertain the specificity of the N-AI effect, we performed parallel experiments by co-transfecting HCT116 cells with pGL3N2PR-2327/-99 and pCGN-HA-S33Y-*β*-catenin, a plasmid encoding an S33Y-mutated *β*-catenin protein known to activate the *NOTCH2* promoter through the Wnt pathway.^33^ Results showed that N-AI treatment did not modify the ability of S33Y-*β*-catenin to activate the *NOTCH2* promoter compared with DMSO-treated cells (Figure 4c). Conversely, experiments with the Wnt signaling inhibitor XAV-939 showed that XAV-939 did not counteract *NOTCH2* promoter induction by TERT compared with DMSO-treated cells (data not shown). These data suggest that NF-*κ*B, but not Wnt signaling, is involved in the TERT-mediated activation of NOTCH2 expression.

To further investigate the involvement of NF-*κ*B signaling in the regulation of the *NOTCH2* promoter by TERT, we performed the luciferase reporter assay with a smaller *NOTCH2* luciferase promoter reporter construct (pGL3N2PR-110), covering the most proximal LEF-1/TCF-site but lacking two putative NF-*κ*B binding motifs.^33, 38^ In agreement with a previous study,^33^ we observed that luciferase activity was similarly increased by co-transfection with pCGN-HA-S33Y-*β*-catenin with either pGL3N2PR-2327/-99 or pGL3N2PR-110 (7.5-fold increase for pGL3N2PR-2327/-99 and 6.6-fold increase for pGL3N2PR-110 compared with co-transfection with the control pcDNA3 plasmid; Figure 4d). In contrast, pEGFP–hTERT activated pGL3N2PR-110 significantly less efficiently than pGL3N2PR-2327/-99 (*P*<0.001), (3.4-fold increase for pGL3N2PR-110 and 6.8-fold increase for pGL3N2PR-2327/-99 compared with co-transfection with the control pEGFP-C1 plasmid; Figure 4d). Taken together, these results demonstrate that TERT activates the *NOTCH2* promoter and that this effect is mostly mediated by the NF-*κ*B signaling pathway.

### Inhibition of NOTCH signaling triggers EBV lytic cycle

**Figure 5:**
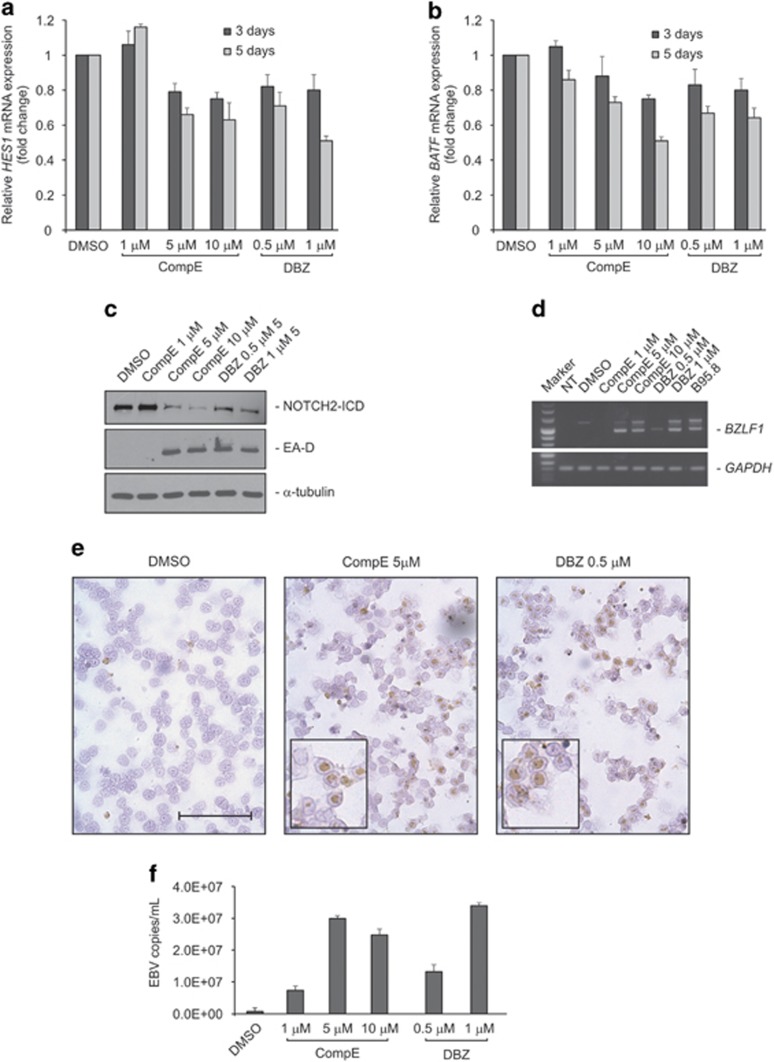
Inhibition of NOTCH signaling induces EBV lytic cycle. 4134/TERT+ cells were treated for 3 and 5 days with indicated concentrations of GSIs (CompE or DBZ) or DMSO as control. *HES1* (**a**) and *BATF* (**b**) transcripts were quantified by real-time PCR. Values are the means and S.D. (bar) of three replicates. (**c**) Expression of lytic EA-D viral protein, cellular NOTCH2 and housekeeping *α*-tubulin was assessed by western blot after 5 days of GSIs treatment. Panels are representative of three separate experiments. (**d**) *BZLF1* (upper panels) and housekeeping *GAPDH* (lower panels) mRNA were analyzed by RT-PCR after 5 days of GSI treatment. Panels are representative of three separate experiments. NT, no treated cells. (**e**) gp350 protein expression in 4134/TERT+ cells at 5 days of treatment with indicated concentration of GSI or with DMSO as control (20x). Scale bar, 100 *μ*m. The inserts were 40x magnification. (**f**) Real-time PCR quantification of EBV DNA in cell culture supernatants after ultracentrifugation and DNase treatment after 5 days of GSI treatment. Values are the means and S.D. (bar) of three replicates

We have previously demonstrated that, in the LCL system, the viral latent membrane protein 1 (LMP1) activates TERT at transcriptional level,^39^ whereas TERT silencing triggers the viral lytic cycle.^9^ This effect is associated with downregulation of BATF, a transcription factor able to modulate *BZLF1* expression. According to these previous findings, we hypothesized that TERT can affect EBV latent/lytic status by modulating NOTCH2 expression which, in turn, may influence BATF expression;^13^ thus, inhibition of NOTCH2 signaling was predicted to induce activation of the EBV lytic cycle. To test this hypothesis, we treated 4134/TERT+ with gamma-secretase inhibitors (GSIs), including compound E (CompE) and dibenzazepine (DBZ). GSIs are compounds that block the final cleavage of the precursor form of NOTCH, thus preventing generation of ICD and inhibiting NOTCH signaling. GSIs treatment of 4134/TERT+ cells reduced both the canonical NOTCH target gene, *HES1* and *BATF* expression in a time- and dose-dependent manner (Figures 5a and b). Western blot analysis confirmed that the cleavage of NOTCH2 was inhibited by treatment with GSIs (Figure 5c). Inhibition of NOTCH signaling at 5 days also resulted in the expression of lytic viral *BZLF1* and *E-AD* genes (Figures 5c and d). GSI treatment consistently resulted in a remarkable increase in the number of positive cells expressing late viral lytic gp350 (from <1% in control cell cultures to ~35% in cell cultures treated with 5*μ*M CompE or 0.5 *μ*M DBZ; Figure 5e). B95.8 cells were employed as positive control for EBV lytic protein expression (Supplementary Figure 1). The complete induction of the viral lytic cycle was confirmed by the release of EBV virions, as shown by increased EBV DNA levels in DNase-treated culture supernatants (Figure 5f) after 5 days of GSI treatment compared with supernatant from untreated control cells.

**Figure 6:**
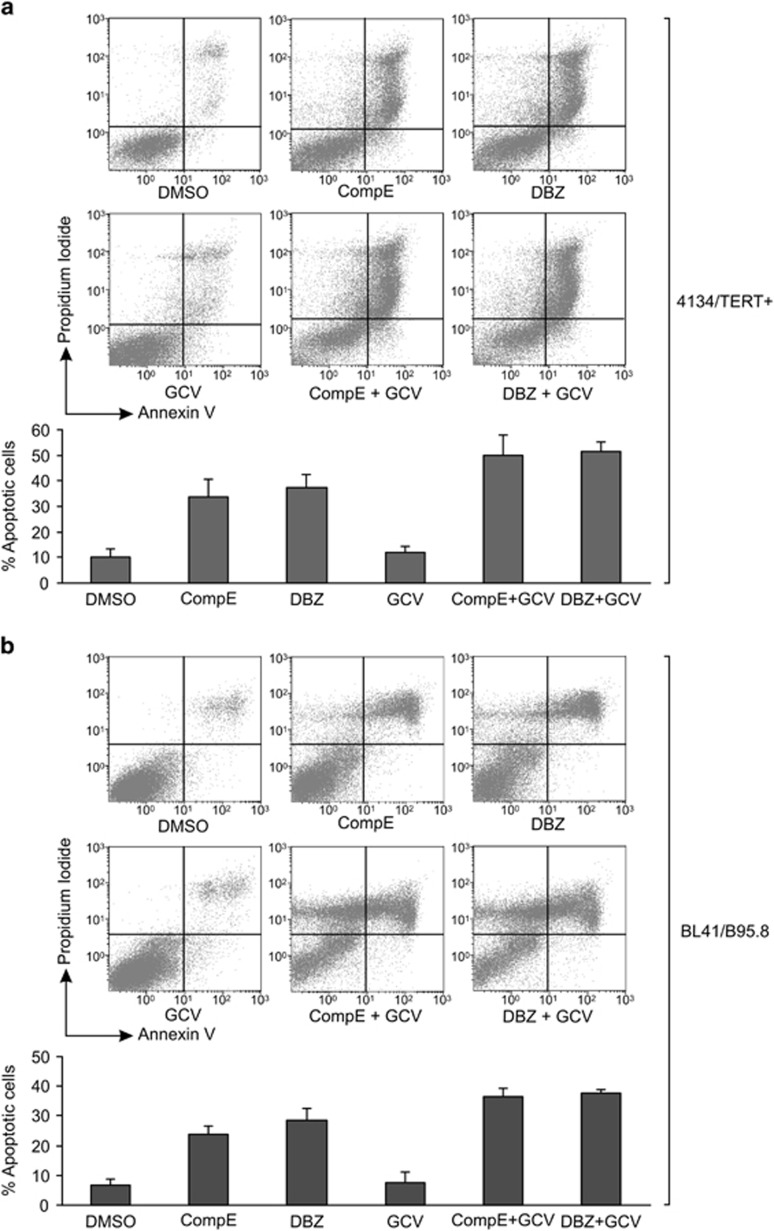
Effect of GSIs and GCV on cell viability. 4134/TERT+ cells (**a**) and BL/41B95.8 cells (**b**) were cultured for 5 days in presence of GSI (5 *μ*M of CompE or 0.5 *μ*M of DBZ) with or without 100 *μ*M of GCV. Cells were labeled with annexin V/PI and analyzed by flow cytometry. Panels are representative of three separate experiments. Percentages of apoptotic cells are shown in the graphs on the bottom. Values are means and S.D. (bar) of three separate experiments

As the EBV lytic cycle promotes the death of infected cells, we studied the possible pro-apoptotic effect of GSI treatment on EBV-infected cells. Treatment of 4134/TERT+ cells for 5 days with 5 *μ*M of CompE or 0.5 *μ*M of DBZ induced an increase in the number of apoptotic cells (by >30%) compared with DMSO-treated cells (Figure 6a). The number of cells undergoing apoptosis after GSI treatment was consistent with the percentage of cells positive for late lytic gp350 viral protein. In agreement with published data,^40^ in EBV-negative BL41 cells, treatment with GSIs (5 *μ*M of CompE or 0.5 *μ*M of DBZ), only slighty increased the percentage of apoptotic cells (Supplementary Figure 2). In EBV- and TERT-negative U2OS cells, treatment with 10 *μ*M of CompE or with 1 *μ*M of DBZ did not alter cell viability compared with untreated control cells (data not shown). Furthermore, in view of the emerging interest in approaches combining lytic cycle inducers with antiviral drugs to treat EBV-driven tumors, we studied the apoptotic effect of combined treatment with GSI and ganciclovir (GCV), an antiviral pro-drug that is activated by viral lytic protein kinase.^41, 42, 43^ As shown in Figure 6a, the combined treatment of 4134/TERT+ cells with GSI and GCV further increased the rates of apoptotic cells compared with cells treated with GSI alone (Figure 6a). However, treatment with GCV alone did not affect cell viability. The increased rate of apoptotic cells in cultures treated with a combination of GCV and GSI compared with GSI treatment alone suggests that in some GSI-treated cells the EBV lytic reactivation is abortive, but sufficient to produce the early lytic EBV protein kinase able to activate the pro-drug GCV.^44^ Lastly, activation of the EBV lytic cycle after GSI treatment was also observed in EBV-positive cells of BL cell line BL41/B95.8 (data not shown). As observed in 4134/TERT+, also in BL41/B95.8 cells GSI treatment induced a pro-apoptotic effect enhanced by combined treatment, GSI with GCV (Figure 6b), indicating that this is a general phenomenon for EBV-carrying B lymphocytes.

## Discussion

Despite their pathogenic importance, the mechanisms underlying EBV reactivation *in vivo* are poorly understood. Available data obtained from *in vitro* models indicate that EBV lytic cycle can be elicited by treatment of latently infected cells with a variety of reagents including 12-*O*-tetradecanoyl-phorbol-1-acetate, calcium ionophores, sodium butyrate, anti-immunoglobulin antibodies and TGF-*β*.^45,46,47^ The effects of all these reagents converge on the upregulation of two EBV immediate–early genes, *BZLF1* and *BRLF1*, which orchestrate the activation of viral lytic replication.^48^ Nevertheless, in all experimental conditions investigated so far, only a fraction of cells treated with these reagents enter the lytic cycle, the remainder of the population being refractory to lytic replication.^49, 50^ Therefore, identification of cellular factors that regulate the balance between latency and lytic replication of EBV is critical for better understanding of the complex interplay between virus and infected cells.

It is well established that TERT activation is a prerequisite for an efficient EBV-driven B-cell immortalization.^2, 3, 4^ Accumulating evidence indicates that TERT may have additional functions, beyond its role in preserving telomere homeostasis.^7, 8^ Our previous studies have demonstrated that high levels of TERT expression in LCLs prevent the induction of the EBV lytic cycle, which is instead triggered by TERT silencing.^3, 9^ However, the fine mechanisms by which TERT levels affect EBV lytic/latent status have not yet been elucidated. In the present study, we demonstrate that high expression of TERT in LCLs induces an increase in levels of NOTCH2 and its target genes, including BATF, an inhibitor of the expression of *BZLF1,* the main EBV lytic cycle inducer. Therefore, our findings show that TERT contributes to preserve EBV latency in B cells mainly through NOTCH2-dependent BATF activation.

BATF is a member of the AP-1/ATF superfamily of basic leucine zipper transcription factors, able to form heterodimers with Jun proteins to bind to AP-1 consensus sites preferentially.^12^ Available data indicate that BATF expression may antagonize the B-cell growth and inhibit pro-apoptotic gene expression in these cells.^12^ EBV nuclear antigen 2 (EBNA2) has been shown to induce BATF immediately after infection of primary B cells.^13^ Notably, EBNA2 could be regarded as a functional homolog of an active NOTCH receptor, due to its ability to be tethered to promoter regions by interaction with the DNA-binding protein RBPJ.^51, 52^ EBNA2 and NOTCH2 appear to be partially interchangeable as regards to their ability to activate target genes and modulate signaling pathways in B-cell lines.^51^ In the absence of EBNA2, NOTCH may transcriptionally upregulate the expression of EBV LMP 2A, which in turn activates the NOTCH pathway in a positive feedback loop.^53, 54^ On these grounds, our results indicate that, in B cells, TERT activates the NOTCH/BATF cascade, a cellular pathway that is functionally hijacked by EBV for critical regulation of the balance between latency and lytic replication, and induction of immortalization.

The role of NOTCH signaling in B-cell lymphomagenesis is not yet clear, and only limited data are available, particularly for EBV-driven lymphomas; nevertheless, dysfunctions of this pathway may be involved in neoplastic development.^15^ In particular, deregulation of NOTCH2 signaling and consequent CD23 upregulation have been observed in B-cell malignancies, such as B-cell chronic lymphocytic leukemia.^55, 20, 21, 22, 23, 24^ Overexpression of NOTCH2 has been found in some marginal zone lymphomas,^25^ and potential activating mutations or mutations resulting in NOTCH2-reducted turnover have also been detected in marginal zone lymphoma and in diffuse large B-cell lymphomas (DLBCL).^25, 26^ More recently, a fraction of DLBCLs were shown to carry a truncated NOTCH2 mutation that leads to partial deletion of the C-terminal PEST domain; this deletion was shown to activate both NOTCH2 and NF-*κ*B signals and to promote the proliferation of B-cell lymphoma cell lines.^27^ Characterization of tumor biopsies from Hodgkin lymphoma patients revealed a strong expression of NOTCH in Hodgkin–Reed–Stenberg tumor cells; activation of NOTCH signaling in these cells promoted proliferation and provided protection against apoptosis.^28, 29, 30^

Here, we found that activation of NOTCH2 signaling has an important role in maintaining a homeostatic equilibrium between B cells and the virus, being capable of keeping a strictly latent EBV cycle. In particular, we demonstrate that inhibition of NOTCH2 signaling by GSIs induces expression of EBV lytic genes and triggers the induction of a complete lytic cycle in both LCL and EBV-positive BL cells. As viral lytic replication is associated with the death of infected cells, this study suggests that, in addition to TERT, NOTCH2 may constitute an important therapeutic target for EBV-driven B-cell malignancies. It has recently been reported that NOTCH2, activated via delta-like ligand 1, inhibits the EBV lytic cycle in the EBV-infected B-cell non-Hodgkin's lymphoma line by upregulating the cellular transcription factor Zeb2, which represses *BZLF1* expression. Inhibition of NOTCH2 signaling led to disruption of EBV latency, with induction of *BZLF1* and the lytic cycle.^56^ Thus, our study provides independent validation of the role of NOTCH2 in the balance between latent and lytic status of EBV in infected B cells, albeit by different mechanisms. The discovery of different mechanisms involved in the regulation of *BZLF1* by NOTCH2 may reflect the complex pathway that ensure EBV latency in B cells.^46^

In this study, we also provide mechanistic insights demonstrating that TERT can induce NOTCH2 expression at transcriptional level. This is consistent with recent studies indicating a telomere-independent role for TERT as a transcriptional modulator of the Wnt/*β*-catenin and NF-*κ*B signaling pathways.^35, 36, 37^ In particular, we found that NF-*κ*B, but not the Wnt signaling, is involved in *NOTCH2* promoter induction by TERT. These results were observed with both an NF-*κ*B signaling inhibitor and a *NOTCH2* luciferase promoter reporter construct lacking two putative NF-*κ*B binding motifs. The involvement of the NF-*κ*B pathway in *NOTCH2* promoter activation was also recently demonstrated by Wang *et al.*^38^ who reported that inflammatory cytokine-dependent induction of NOTCH2 in nucleus pulposus cells requires direct interaction of the NF-*κ*B/p65 protein with the NOTCH2 promoter.

Overall, the results of the present study provide new insights into how cellular genes coordinately control EBV latency. In particular, we demonstrate that TERT contributes to preserve EBV latency through the NOTCH2 cellular pathway; as virus latency is required for EBV-driven cell transformation, this study suggests that NOTCH2 has a significant role in LCL immortalization. Our finding that NOTCH2 inhibition triggers the EBV lytic cycle and cell apoptosis is of particular importance in the light of increasing interest in developing strategies to reactivate EBV lytic gene expression in latently infected tumor cells to treat overt EBV-associated lymphomas.^57, 58, 59^ Several chemotherapeutic drugs are known to trigger EBV replication, and the combination of antivirals with lytic cycle inducers is emerging as a highly promising strategy for the treatment of EBV-driven tumors.^60, 61^ On these grounds, our results also demonstrate that the antiviral drug GCV can enhance the apoptotic effect induced by GSI treatment in both LCL and EBV-positive BL. Our findings therefore suggest that GSIs can be combined with other drugs in therapeutic schedules aimed at inducing EBV lytic reactivation against EBV-associated lymphomas. In this respect, it will be of interest in future studies to assess whether TERT inhibitors^9^ can synergize with GSIs.

## Materials and Methods

### Plasmids

Plasmids were provided as follows: a plasmid expressing luciferase under the control of *NOTCH2* promoter (pGL3N2PR-2327/99), a smaller *NOTCH2* reporter plasmid lacking two putative NF-*κ*B binding motifs (pGL3N2PR-110) and a plasmid expressing an S33Y-mutated *β*-catenin protein (pCGN-HA-S33Y-*β*catenin)^33^ obtained from Jonas Ungerback (Linkopings University, Linkoping, Sweden); human TERT linked to EGFP in pEGFP (pEGFP–hTERT) and the control empty vector pEGFP-C1^34^ obtained from Chantal Autexier (Lady Davis Institute for Medical Research, Jewish General Hospital, Montréal, Québec, Canada); plasmids expressing the intracellular domain of NOTCH2 (pMigRI-ICN2 and pMSCVpuro-ICN2) from Adolfo Ferrando (Columbia University, New York, NY, USA); plasmid containing 800- bp fragment upstream of the TERT translational start site, phTERTpromoterLuc^62^ from Riccardo Dalla Favera (Columbia University).

### Cell cultures

LCLs 4134, 4810, 4193, 4815 and 4141 were obtained by infecting peripheral blood mononuclear cells from normal donors with EBV strain B95.8. The establishment and characterization of these cell lines have been previously described.^3^ We considered as ‘early LCLs' cells within the first 30 culture passages after EBV infection, and ‘late LCLs' cell lines which underwent up to 90 culture passages after EBV infection.^3^ Cells (4134) expressing ectopic TERT (4134/TERT+) and control cells (4134/BABE) were obtained by infecting parental telomerase-negative cells with the pBABE retroviral vector, either containing or lacking TERT cDNA, respectively.^3^ 4134/MigRI-ICN2 and 4134/MSCVpuro-ICN2 cells were obtained by infecting 4134 cells with retroviral vectors expressing the NOTCH2-ICD (pMigRI-ICN2 or pMSCVpuro-ICN2). Retroviral vectors were generated by a transient three-plasmid vector packaging system, as previously described.^3^ BL41 is an EBV-negative BL cell line, kindly provided by Martin Rowe (Cancer Center, University of Birmingham, Birmingham, UK). BL41/B95.8 is the counterpart cell line infected *in vitro* with EBV strain B95.8 and was kindly provided by Martin Allday (Ludwig Institute for Cancer Research, London, UK). B95.8 cell line is an EBV-positive marmoset lymphoblastoma cell line, which spontaneously has around 5% of cells in the lytic cycle,^63^ was employed as positive control for lytic protein expression. LCLs and BL41 cells were cultured in RPMI-1640 medium (Euroclone, Milano, Italy), supplemented with 2% glutamine, 50 *μ*g/ml gentamycin (Sigma, St Louis, MO, USA) and 10% heat-inactivated fetal bovine serum (FBS; Gibco, Milano, Italy; standard medium). BL41/B95.8 and B95.8 cells were cultured in standard medium supplemented with 1 mM sodium pyruvate, 1% nonessential amino acids (Sigma), and 50 mM *β*-mercaptoethanol. HCT116 and U2OS cells were obtained from the American Type Culture Collection (Rockville, MD, USA) and maintained in McCoy's 5 A modified medium (Sigma) supplemented with 50 *μ*g/ml gentamycin (Sigma) and 10% heat-inactivated FBS (Gibco). All cell lines were maintained in culture at 37 °C and 5% CO_2_.

To inhibit NOTCH signaling, cells in logarithmic growth were cultured in the presence of compound E (CompE, EDM Millipore, Billerica, MA, USA) or dibenzazepine (DBZ, EDM Millipore) at different doses with or without the pro-drug ganciclovir (GCV; Sigma). Mock-treated cells were cultured in the presence of a vehicle (DMSO) at a final concentration not exceeding 0.1%.

### Reverse transcriptase PCR and real-time PCR

Total cellular RNA was extracted with TRIzol reagent (Invitrogen, Carlsbad, CA, USA). For reverse transcriptase PCR (RT-PCR) and real-time PCR experiments, 1-*μ*g RNA was retro-transcribed into cDNA with the SuperScript III RNase reverse transcriptase assay (Invitrogen) according to the manufacturer's instructions. All *TERT* transcripts were quantified by real-time PCR, as previously described.^3^ Real-time PCR reactions for quantification of *BATF* and *NOTCH2* transcripts were performed on a LightCycler 480 Real-Time PCR System (Roche, Basel, Switzerland). Each PCR was conducted in 50 *μ*l of mixture containing 25 *μ*l Lightcycler 480 probe master (Roche), 200 nM of fluorogenic probe, 900 nM of each primer and 10 *μ*l of cDNA samples. After 2 min at 50 °C to allow the uracil *N*-glycosylase to act, and after a denaturation step of 10 min at 95 °C, 45 cycles were run, each for 15 s at 95 °C and 1 min at 60 °C. Samples were run in triplicate. Primers and probes for PCR analysis were: BATF forward: 5′-GACAAGAGAGCCCAGAGGTG-3′ BATF reverse: 5′-GTAGAGCCGCGTTCTGTTTC-3′ BATF probe: 5′-Cy5-TGGCAAACAGGACTCATCTG-BBQ-3′ NOTCH2 forward: 5′-CAGCCTGTATGTGCCCTGTG-3′ NOTCH2 reverse: 5′-GTGCTCCCTTCAAAACCTGGA-3′ NOTCH2 probe: 5′-FAM-TCACCTTGTGTCAATGGAGGC-BHQ-3′. Quantification of *HES1* and *JAG1* transcripts were carried out by real-time PCR on an ABI PRISM 7900HT Sequence Detection System (PE Biosystems, Foster City, CA, USA) by using Platinum SYBR Green qPCR SuperMix-UDG (Invitrogen) and the following primers: HES1 forward: 5′-CAGCGGGCGCAGATGAC-3′ HES1 reverse: 5′-CGTTCATGCACTCGCTGAAG-3′ JAG1 forward: 5′-TGAATGGGGGTCACTGTCAGA-3′ JAG1 reverse: 5′-CACCGTTCTGGCAGGGATTAG-3′. Cycling conditions were 10 min at 95 °C, 60 cycles of 15 s at 95 °C and 1 min at 60 °C. For mRNA normalization, 10 *μ*l of cDNA from each sample were amplified for the hypoxanthine phosphoribosyltransferase 1 (*HPRT1*) housekeeping gene, as described previously.^64^ *TERT* values were normalized for 10^4^ copies of *HPRT1*. Relative quantification for *NOTCH2*, *BATF, HES1* and *JAG1* expression was carried out with the ΔΔ*C*t method and *HPRT1* as reference gene, unless otherwise specified.

*NOTCH2* transcripts were detected by the specific primer pair designed in the ICD region: NOTCH2 forward: 5′-CTGGATGCAGGTGCAGATGCCAATGC-3′ and NOTCH2 reverse; 5′-GCAGAAGTCAACACGGTGCCTGGAGG-3′.^55^ Viral *BZLF1* and cellular housekeeping glyceraldehyde-3-phosphate dehydrogenase (*GAPDH*) gene transcripts were detected, as previously reported.^65^

All primer pairs of *NOTCH2*, *BATF, HES1, JAG1, TERT, GAPDH* and *HPRT1* cellular genes were designed in different exons, separated by at least one intron to avoid genomic DNA amplification during PCR reactions. Nevertheless, to account for potential contamination by genomic DNA, control reactions without reverse transcriptase (RT−) were included in each plate. All RT− samples were negative, as expected (not shown).

### Telomerase activity assay

Telomerase activity was assessed with a PCR-based telomeric repeats amplification protocol (TRAP) as previously reported.^66, 67^ The TRAP assay was performed with 0.250 *μ*g of total cell lysates.

### Western blot

Western blot analyses were prepared as previously reported.^68^ The expression of viral EA-D and cellular NOTCH2-ICD, BATF, JAG1 and *α*-tubulin were evaluated by monoclonal anti-EA-D (clone 6D1, Abcam, Cambridge, UK), monoclonal anti-NOTCH2 (clone C651.6DbHN developed by Spyros Artavanis-Tsakonas, obtained from the Developmental Studies Hybridoma Bank, developed under the auspices of the NICHD and maintained by The University of Iowa, Department of Biology, Iowa City, IA, USA), monoclonal anti-BATF (1G4; Novus Biologicals, Cambridge, UK), polyclonal anti-JAG1 (H114; Santa Cruz Biotechnology, Dallas, TX, USA) and clone B-512 (Sigma), respectively. Blots were incubated with an appropriate peroxidase-conjugated secondary antibody (Sigma), and stained with a chemiluminescence detection kit (SuperSignal West Pico Chemiluminescent Substrate, Pierce, Rockford, IL, USA).

### Immunohistochemistry

Cytospins were fixed in cold acetone (4 °C) for 10 min. Expression of gp350 protein was analyzed using the monoclonal antibody clone 0221 (Abcam). After incubation for 1 h with the primary antibody, immunostaining was performed with the avidin–biotin peroxidase complex (ABC kit; Vector Laboratories, Burlingame, CA, USA), and 3–30 diaminobenzidine chromogen as substrate (Dako, Glostrup, Denmark). The cells were lightly counterstained with Mayer's hematoxylin. The specificity of the staining procedure was confirmed by replacing the primary antibody with PBS.

### Luciferase reporter assay

HCT116 cells (4.5 × 10^5^) were transiently transfected with 1 *μ*g of pGL3N2PR-2327/-99, serial dilution of pEGFP–hTERT, or empty pEGFP-C1 and 0.5 *μ*g cytomegalovirus β-galactosidase (pCMV-β-Gal). A set of experiments were also conducted with a smaller *NOTCH2* luciferase promoter reporter construct (pGL3N2PR-110) instead of the longer pGL3N2PR-2327/-99. In some experiments, cells were treated with the inhibitors XAV-939 (Selleck Chemicals LLC, Houston, TX, USA) or N-AI (EDM Millipore) at the indicated concentration 3 h before transfection. DMSO was employed as control. As positive control, HCT116 were also co-transfected with pCGN-HA-S33Y-*β*-catenin a plasmid known to activate the *NOTCH2* promoter or with pcDNA3 as control plasmid. Lipofectamine 2000 (Invitrogen) was used as a transfection reagent according to the manufacturer's instructions.

TERT-negative U2OS cells (3x10^5^) were transiently transfected with 1.5 *μ*g phTERTpromoterLuc, 1 *μ*g pMT2TMyc, or empty pMT2T, 1 *μ*g pMigRI-ICN2 or pMSCVpuro-ICN2, and 0.5 *μ*g CMV- β-Gal by Lipofectamine 2000 (Invitrogen). Luciferase and β-Gal activities were quantified with the Bright-Glo luciferase assay and Beta-Glo system (Promega, Madison, WI, USA), respectively. Luciferase activity was normalized to β-galactosidase activity and the data are presented as luciferase activity compared with that of the control vector.

### EBV genome quantification

EBV DNA from virus particles released in culture supernatants were quantified by real-time PCR^69^ after ultracentrifugation and DNase treatment, as previously reported.^3^

### Apoptosis analysis

Apoptosis was evaluated by staining cells with annexin V and propidium iodide (PI, Roche), as previously detailed,^68^ and analyzed by flow cytometry. At least 50 000 events were acquired, data were processed with CellQuestPro software (BD Biosciences, Erembodegem, Belgium), and analyzed with Kaluza Analyzing Software v1.2 (Beckman Coulter, Brea, CA, USA). Annexin V-positive/PI-negative and annexin V-positive/PI-positive samples were classified as early and late apoptotic cells, respectively, and both fractions were included in the percentages of apoptotic cells.

### Statistical analysis

For statistical comparisons, the Student's *t*-test or the Mann–Whitney *U* test were conducted with SPSS software version 21 (IBM, Armonk, NY, USA). *P-*value <0.05 was considered to be statistically significant.

## Abbreviations

EBV: Epstein–Barr virus
LCL: lymphoblastoid cell line
TERT: telomerase reverse transcriptase
EA-D: EBV early antigen diffuse
gp350: EBV glycoprotein 350
BL: Burkitt lymphoma
BATF: basic leucine zipper transcription factor, ATF-like
NOTCH2-ICD: NOTCH2 intracellular domain
JAG1: jagged 1
HES1: hes family bHLH transcription factor 1
MYC: v-myc avian myelocytomatosis viral oncogene homolog
DMSO: dimethylsulfoxide
N-AI: NF-*κ*B activation inhibitor
LMP1: latent membrane protein 1
EBNA2: EBV nuclear antigen 2
GSI: gamma-secretase inhibitor
CompE: compound E
DBZ: dibenzazepine
GCV: ganciclovir
DLBCL: diffuse large B-cell lymphoma
EGFP: enhanced green fluorescent protein
HPRT1: hypoxanthine phosphoribosyltransferase 1
GAPDH: glyceraldehyde-3-phosphate dehydrogenase
TRAP: telomeric repeats amplification protocol
TA: telomerase activity
