# Supplementary material for: Cross talk between EBV and telomerase: the role of TERT and NOTCH2 in the switch of latent/lytic cycle of the virus
Source: Cell Death Dis. 2015 May 28;6(5):e1774–. doi: 10.1038/cddis.2015.145 (PMC4669716; doi:10.1038/cddis.2015.145)
Supplement: Supplementary Figure Legends [file cddis2015145x1.doc]

**LEGENDS TO SUPPLEMENTARY FIGURES**

**Supplementary Figure 1.** Untreated and treated B95.8 cells were analyzed for lytic EBV protein expression. B95.8 cells were treated with a combination of TPA (20 ng/mL final concentration) and sodium butyrate (3 mM final concentration) for 48 hours (TPA+NaB). NT: non-treated cells. (A) Expression of viral EA-D and cellular α-tubulin as loading control was assessed by western blot. (B) gp350 protein expression was assessed by immunohistochemistry.20x magnification. Scale bar, 100 µm. Inserts were 40x magnification of the dotted boxes. ~5% of untreated- and > 50% treated- B95.8 cells are gp350-positive.

**Supplementary Figure 2.** BL41 cells were cultured for 5 days in presence of GSI (5 µM of CompE or 0.5 µM of DBZ). Cells were labeled with annexin V/PI and analysed by flow cytometry. Percentages of apoptotic cells are shown in the graph. Values are means ad SD (bar) of 3 replicates. GSI-treated cells show a slight increase of apoptotic cells compared with DMSO-treated control cells.
