# Supplementary figures and images for: Cross talk between EBV and telomerase: the role of TERT and NOTCH2 in the switch of latent/lytic cycle of the virus
Source: Cell Death Dis. 2015 May 28;6(5):e1774–. doi: 10.1038/cddis.2015.145 (PMC4669716; doi:10.1038/cddis.2015.145)

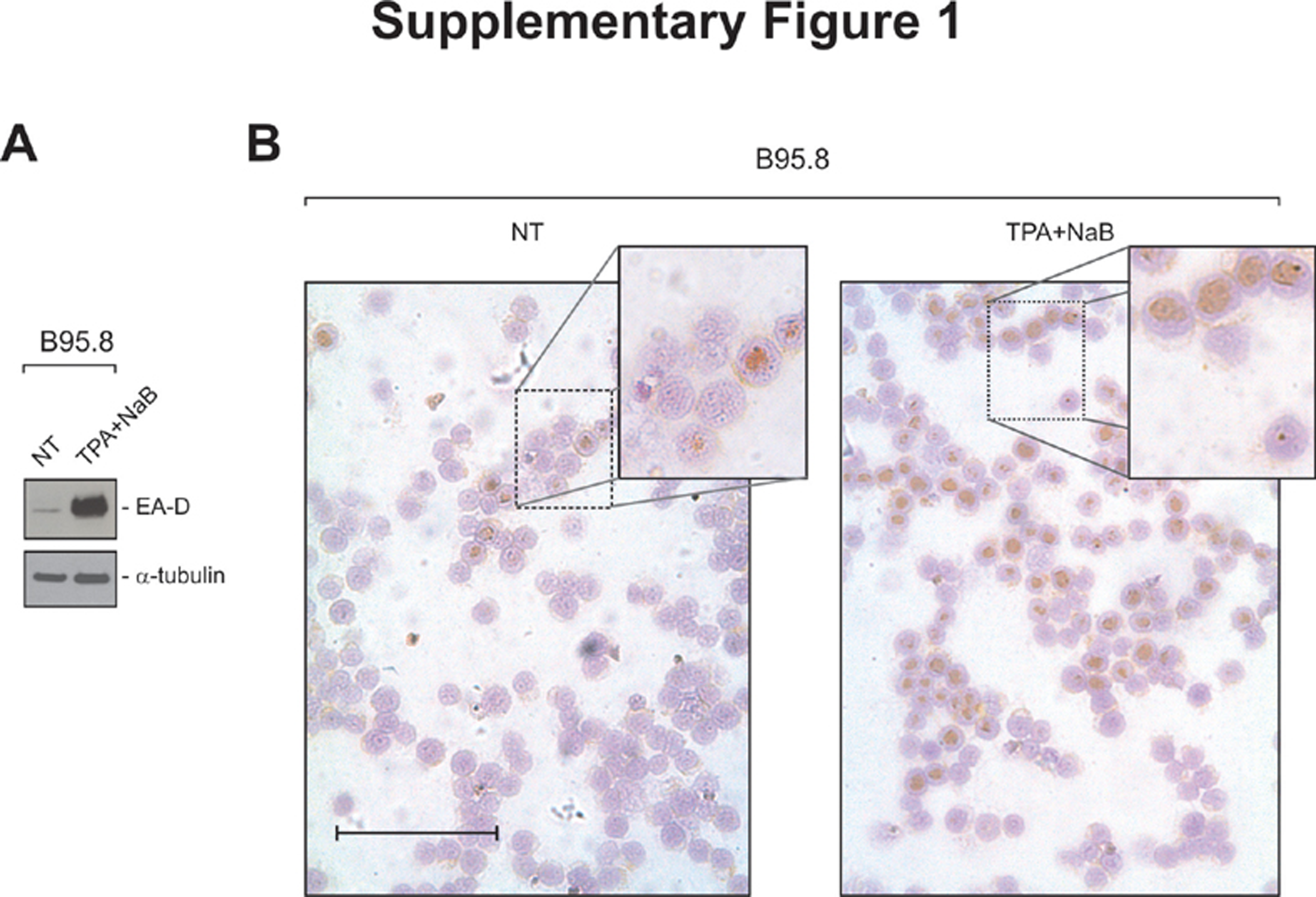

Supplement: Supplementary Figure 1 [file cddis2015145x2.tif]

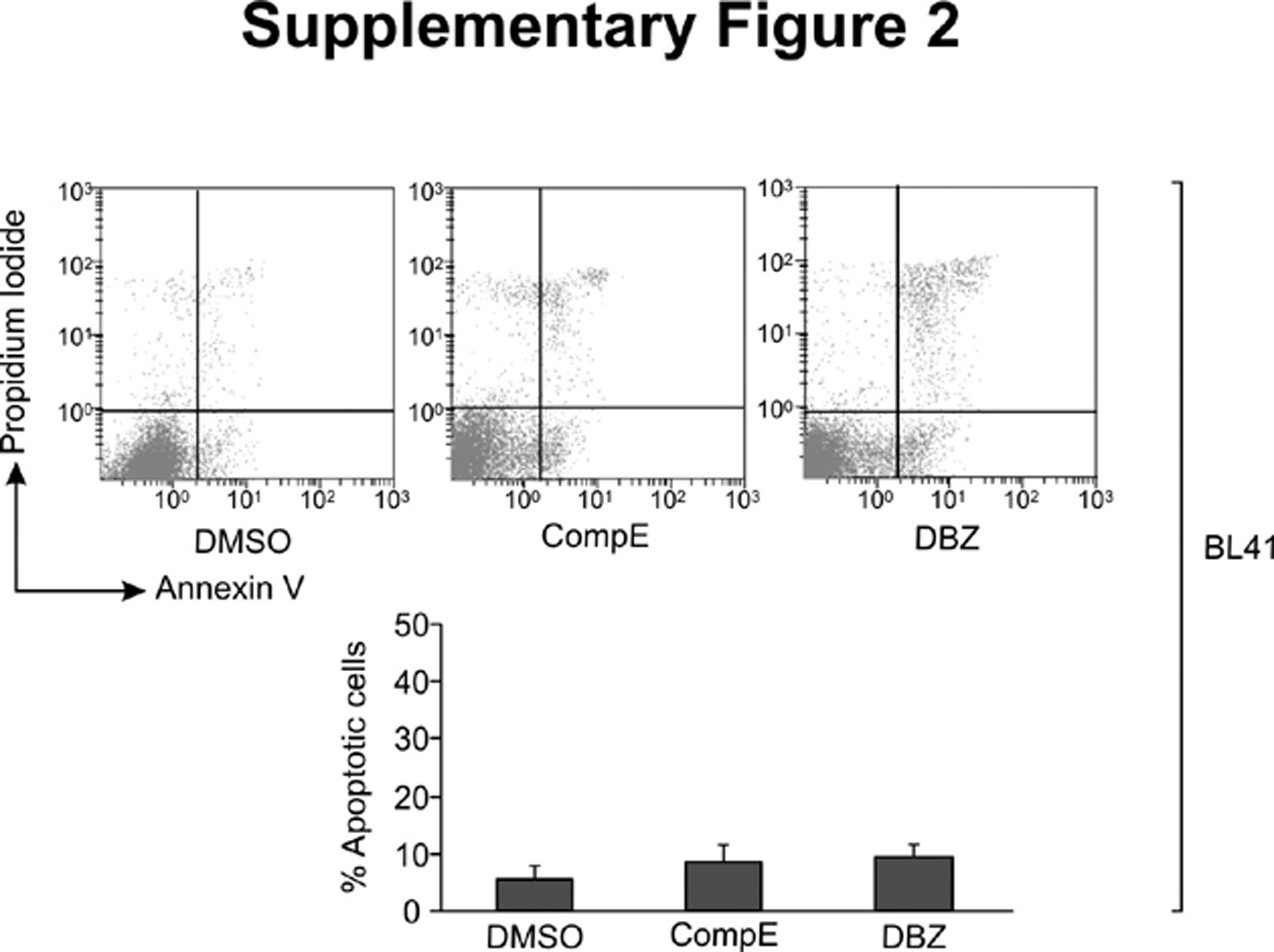

Supplement: Supplementary Figure 2 [file cddis2015145x3.tif]
